# A pan-cancer analysis of the oncogenic role of Holliday junction recognition protein in human tumors

**DOI:** 10.1515/med-2022-0423

**Published:** 2022-02-16

**Authors:** Rong Su, Hechen Huang, Xingxing Gao, Yuan Zhou, Shengyong Yin, Haiyang Xie, Lin Zhou, Shusen Zheng

**Affiliations:** Division of Hepatobiliary and Pancreatic Surgery, Department of Surgery, The First Affiliated Hospital, Zhejiang University School of Medicine, Hangzhou 310003, China; NHC Key Laboratory of Combined Multi-organ Transplantation, Hangzhou 310003, China; Key Laboratory of the Diagnosis and Treatment of Organ Transplantation, Research Unit of Collaborative Diagnosis and Treatment for Hepatobiliary and Pancreatic Cancer, Chinese Academy of Medical Sciences, Hangzhou 310003, China; Key Laboratory of Organ Transplantation, Research Center for Diagnosis and Treatment of Hepatobiliary Diseases, Zhejiang Province, Hangzhou 310003, China; Division of Hepatobiliary and Pancreatic Surgery, Department of Surgery, The First Affiliated Hospital, Zhejiang University School of Medicine, #79 Qingchun Road, Hangzhou 310003, China

**Keywords:** Holliday junction recognition protein, pan-cancer, prognosis, carcinogenesis, cell cycle, nomogram

## Abstract

Although cell-based or animal-based research evidence support the association of Holliday junction recognition protein (HJURP) with cancers, no pan-cancer investigation has been reported. The datasets of Gene Expression Omnibus database along with The Cancer Genome Atlas project were used to evaluate the expression of HJURP in various types of tumors. HJURP is overexpressed in a considerable number of cancers, and some changes in DNA methylation and genetic alterations are discovered in some types of tumors, such as kidney-related and adrenal gland-related tumors. Based on PrognoScan and gene expression profiling interactive analysis (GEPIA), the elevated expression of HJURP worsened the survival time of individuals with cancer. The biological general repository for interaction datasets (BioGRID) and The database for annotation, visualization and integrated discovery (DAVID) were used to establish the functional molecular network. It revealed that the cell cycle and p53 signaling pathway are the key molecular mechanisms that HJURP promotes carcinogenesis. The nomograms between HJURP and clinical pathological factors based on the Cox proportional hazards model showed a good prognostic performance in kidney carcinoma, hepatocellular carcinoma, and lung adenocarcinoma. Our first pan-cancer study provides a relatively profound insights into the oncogenic roles of HJURP across different tumors.

## 1 Introduction

The pan-cancer analysis provides a unique and comprehensive understanding of how, where, and why cancers appear in humans. Due to the complexity of tumorigenesis, the pan-cancer expression analysis helps to assess the association between special genes and clinical outcome/potential molecular mechanism. The Cancer Genome Atlas (TCGA) project and the Gene Expression Omnibus (GEO) database, which contain public functional genomics data of different tumors, allow researchers to perform pan-cancer analysis [1,2].

Holliday junction recognition protein (HJURP) is a histone H3 chaperone that mediates centromere protein A (CENP-A) deposition at human centromeres during the early G1 phase. HJURP is required for cell cycle-specific targeting of CENP-A to centromeres participating in tumorigenesis [3,4]. Highly expressed HJURP gene expression and its transcriptional signature are reported in many cancer types, such as hepatocellular carcinoma, glioma, breast cancer, and bladder cancer [5,6,7]. Our earlier study has already demonstrated that active HJURP promoted cell proliferation through the ubiquitination and cytoplasmic localization of cyclin dependent kinase inhibitor 1A via the mitogen-activated protein kinases1/2 and AKT serine/threonine kinase 1/glycogen synthase kinase 3 beta pathways in hepatocellular carcinoma [8].

Herein, we (1) investigated the association between HJURP and clinical prognosis across TCGA cancers; (2) compared HJURP expression in cancer vs. normal tissues; (3) identified key genomic features such as DNA methylation, mutation, and copy number variation; and (4) described the integrated network and pathway of HJURP.

## 2 Materials and methods

The mRNA expression of HJURP in different types of cancers of microarray datasets was evaluated in the ONCOMINE data resource (www.oncomine.org). Moreover, the mRNA expression of HJURP in different types of cancers in TCGA datasets was evaluated in the tumor immune estimation resource (TIMER) data resource (cistrome.org/TIMER). The methylation, mutation, and copy number variation of HJURP were obtained from cBioPortal (www.cbioportal.org) [9,10,11].

### 2.1 Survival analysis performed in PrognoScan and GEPIA

The association of HJURP with survival in pan-cancer was evaluated in PrognoScan (http://dna00.bio.kyutech.ac.jp/PrognoScan/index.html), as well as GEPIA (http://gepia.cancer-pku.cn) data resources [12,13]. PrognoScan screens the connection linking gene expression to aspects of prognosis of patients, entailing disease-free survival (DFS) and overall survival (OS), over an extensive collection of publicly accessible cancer microarray datasets. The threshold was adjusted to Cox *p*-value <0.05.

### 2.2 Integrated network analysis of HJURP

Integrated network analysis of HJURP was obtained from BioGRID 4.2 (https://thebiogrid.org), consisting of physical and genetic interaction data [14]. Yellow means the line is based on physical interactions. Green means the line is generated from genetic interaction. Purple means colocalization. Node size stands for its weight in the network.

### 2.3 Pathway enrichment analysis

The 50 genes with the highest correlation with HJURP expression were obtained from GEPIA. Pathway enrichment analysis (gene ontology and kyoto encyclopedia of genes and genomes) of HJURP-related genes was performed using DAVID 6.8 [15]. Pathways with a *p*-value threshold of 0.05 were regarded to be significantly regulated.

### 2.4 Prognostic analysis

The multivariate prognostic analysis is expressed by nomogram, based on rms (R package).


**Ethics approval and consent to participate:** Not applicable.

## 3 Results

### 3.1 The mRNA expression and genomic features

**Figure 1:**
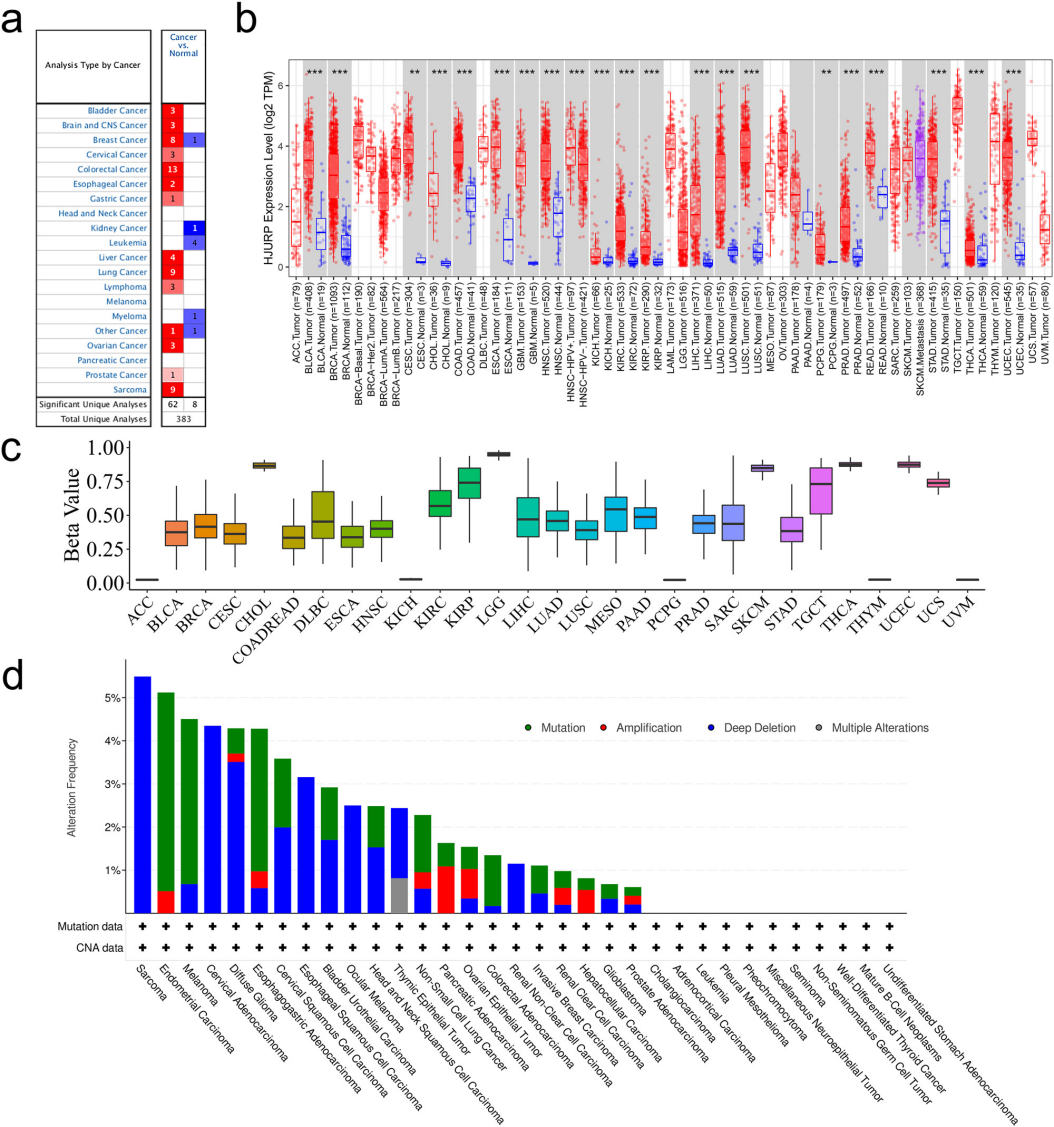
HJURP expression and mutation landscape: (a) In the ONCOMINE database, the expression of HJURP in tumor tissues compared with normal tissues. The number in each unit is the number of data sets. Red means that the tumor is significantly high in tumor tissues; blue is the opposite. (b) The expression level of HJURP of different tumor types in the TIMER database. (c) Boxplot of the methylation levels. (d) HJURP (single mutation) mutation and copy number aberrations in all TCGA cohorts.

As many studies have suggested that HJURP is highly expressed in cancer, it may constitute a pivotal novel target or a biomarker for diagnosis. In the ONCOMINE database, we evaluated 20 kinds of tumors with adjacent normal tissues, showing that 62 out of 383 data sources of microarray included significantly high expression levels of HJURP (Figure 1a). RNA sequencing data in TCGA examined by TIMER showed that the levels of HJURP expression were remarkably higher in tumor tissues in contrast with normal tissues, among BLCA, BRCA, CESC, CHOL, COAD, ESCA, GBM, HNSC, KICH, KIRC, KIRP, LIHC, LUAD, LUSC, PCPG, PRAD, READ, STAD, THCA, and UCEC (Figure 1b). Figure 1c indicates the DNA methylation levels among 29 kinds of tumors, demonstrating the lowest DNA methylation level of HJURP in the kidney-related and adrenal gland-related tumors. Low DNA methylation level may be one of the possible mechanisms that promote the high expression biological effects of the oncogene. To find out the genomic features of HJURP, we checked the genetic alterations in the cBioPortal database. The deep deletion of HJURP was the most accumulated factor in sarcoma, cervical adenocarcinoma, diffuse glioma, and esophageal squamous cell carcinoma. Besides, the mutation frequencies of HJURP are the highest in endometrial carcinoma, melanoma, esophagogastric adenocarcinoma, cervical squamous cell carcinoma, and bladder urothelial carcinoma (Figure 1d).

### 3.2 Multifaceted clinical prognosis of HJURP in cancers

**Figure 2:**
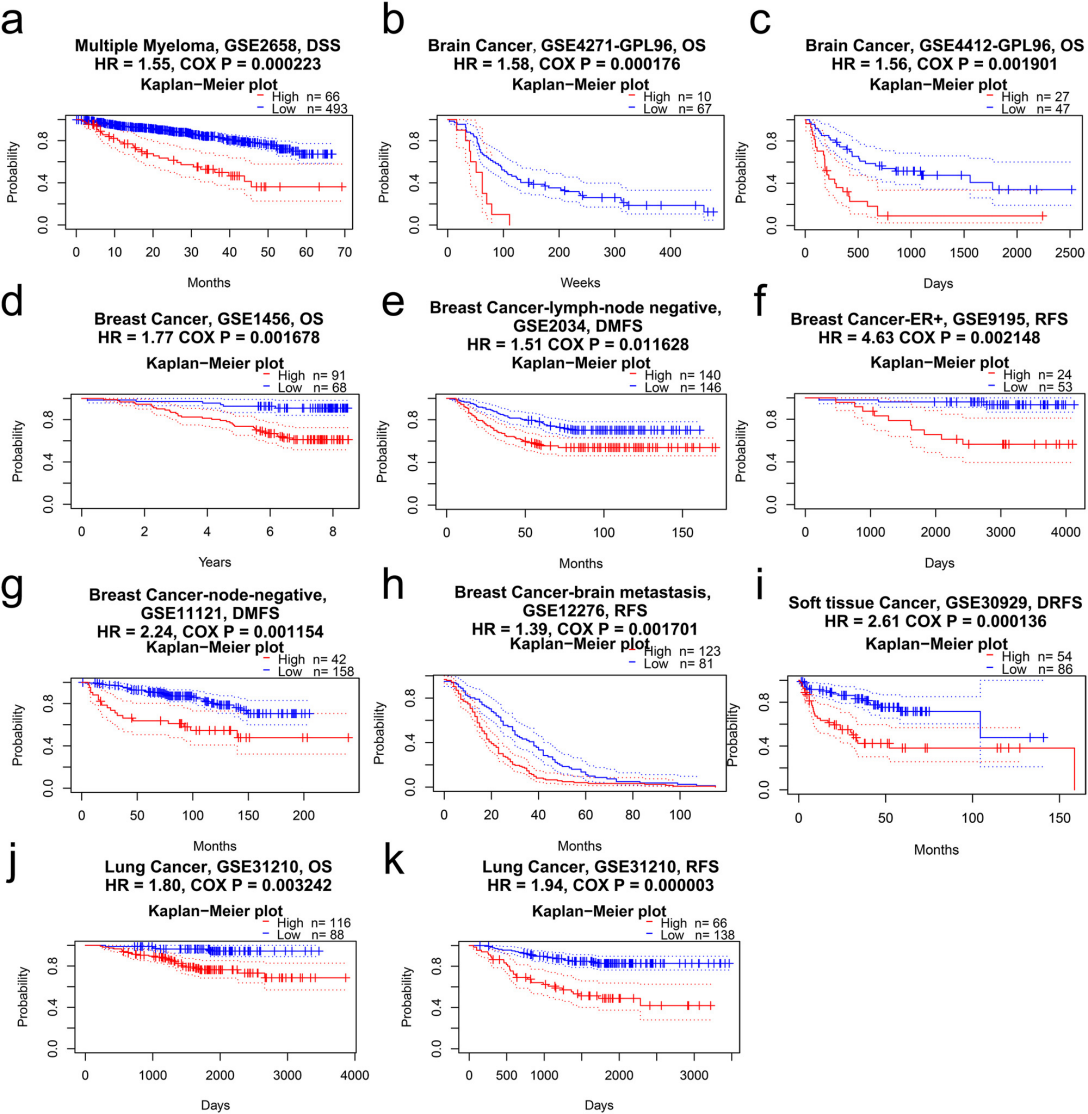
Kaplan–Meier survival curves comparing high and low expression of HJURP in different cancer types in PrognoScan: (a) DSS (*n* = 562) in multiple myeloma cohort GSE2658, (b) OS (*n* = 77) in brain cancer cohort GSE4271, (c) OS (*n* = 74) in brain cancer cohort GSE4412, (d) OS (*n* = 159) in breast cancer cohort GSE1456, (e) DMFS (*n* = 286) in breast cancer cohort GSE2034, (f) RFS (*n* = 78) in breast cancer cohort GSE9195, (g) DMFS (*n* = 200) in breast cancer cohort GSE11121, (h) RFS (*n* = 204) in breast cancer cohort GSE12276, (i) DRFS (*n* = 140) in soft tissue cancer cohort GSE30929, and (j and k) OS and RFS (*n* = 204) in lung cancer cohort GSE31210.

Next, we explored the clinical prognosis of HJURP for pan-cancer in two data resources. In PrognoScan, HJURP expression was remarkably linked to five kinds of cancers, consisting of blood, brain, breast, soft tissue, and lung cancer (Figure 2). HJURP played an oncogenic role in multiple myeloma (GSE2658: DSS, *n* = 559, HR = 1.55, Cox *p* = 0.000223), in brain cancer (GSE4271: OS, *n* = 74, HR = 1.56, Cox *p* = 0.001901; GSE4412: OS, HR = 1.56, Cox *p* = 0.001901), in soft tissue cancer (GSE30929: DRFS, *n* = 140, HR = 2.61, Cox *p* = 0.000136), and in lung cancer (GSE31210: OS, *n* = 74, HR = 1.56, Cox *p* = 0.001901; RFS, HR = 1.94, Cox *p* = 0.000003). HJURP had a very detrimental role in breast cancer (GSE1456: OS, *n* = 159, HR = 1.77, Cox *p* = 0.001678; GSE2034: DMFS, *n* = 286, HR = 1.51, Cox *p* = 0.011628; GSE9195: RFS, *n* = 77, HR = 4.63, Cox *p* = 0.002148; GSE11121: DMFS, *n* = 200, HR = 2.24, Cox *p* = 0.001154; GSE12276: RFS, HR = 1.39, Cox *p* = 0.001701).

**Figure 3:**
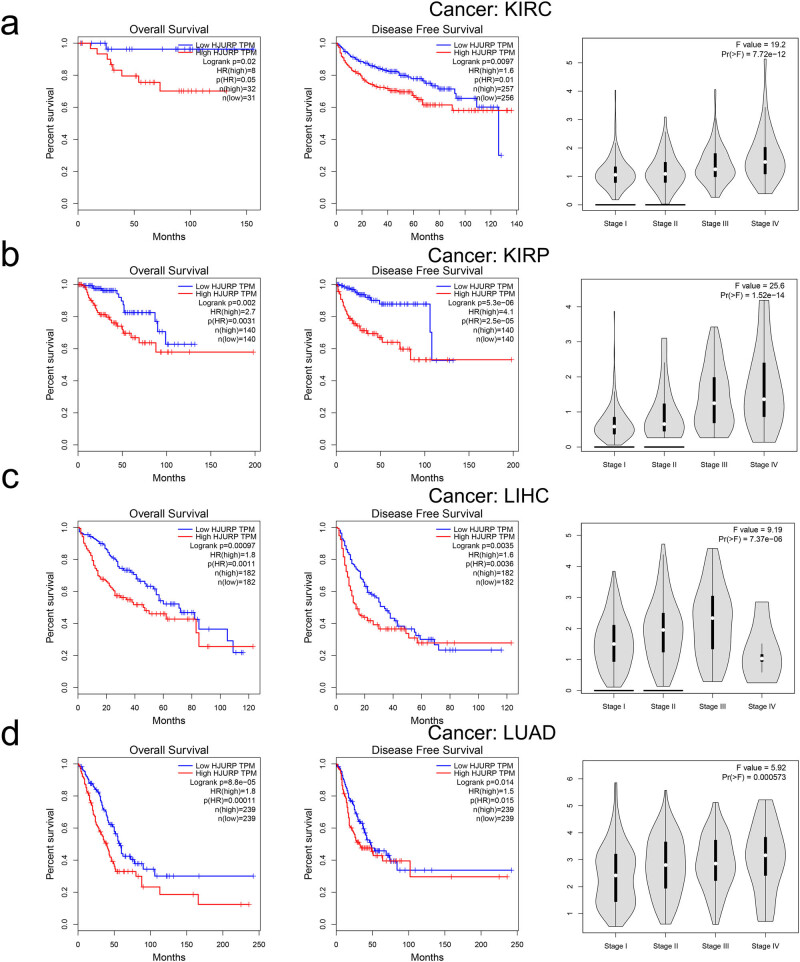
The relationship between the expression of HJURP and clinical prognosis/tumor stage. Kaplan–Meier survival curves and boxplot showed the association between expression of HJURP and OS, RFS and clinical stage of (a) kidney renal clear cell carcinoma, (b) kidney renal papillary cell carcinoma, (c) liver hepatocellular carcinoma, and (d) lung adenocarcinoma. Boxplot “boxes” indicate the first, second, and third quartiles of the data.

**Figure A1:**
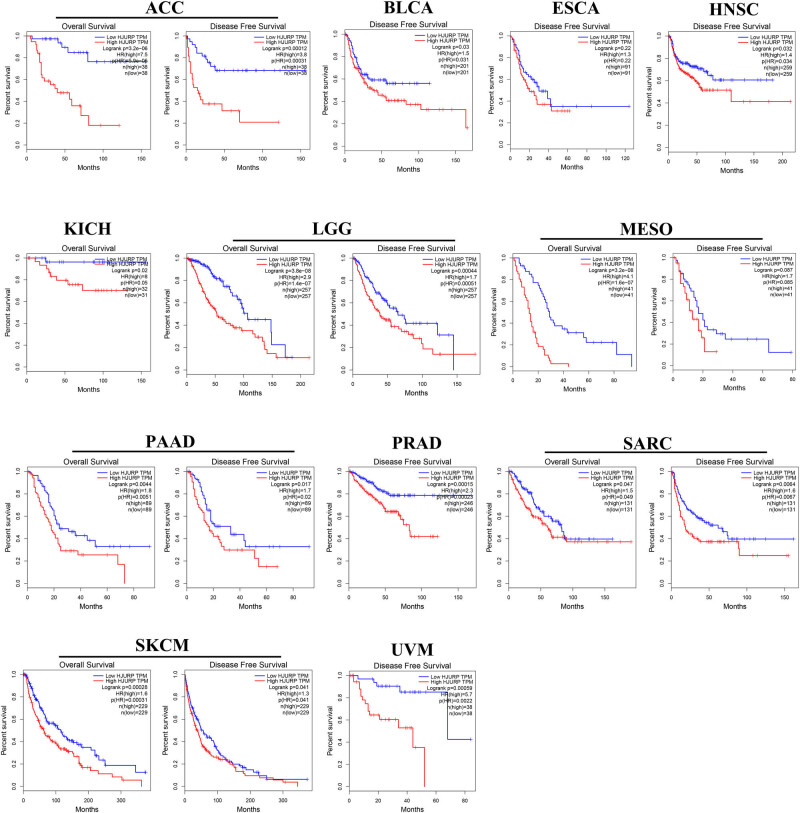
The relationship between the expression of HJURP and clinical prognosis in TCGA.

We further investigated the prognostic value (OS and RFS) of HJURP for pan-cancer in TCGA. In general, HJURP was a harmful index for cancer outcome in most cancer types (Figure 3 and Figure A1). High levels of HJURP expression were harmful for kidney-related cancer (Figure 3a and b). In addition, we indicated that the level of HJURP expression had a forceful positive relationship with patient pathological stages (*p* < 0.001). We also found some similar effects in LIHC and LUAD (Figure 3c and d).

### 3.3 Integrated network and pathway analysis of HJURP

**Figure 4:**
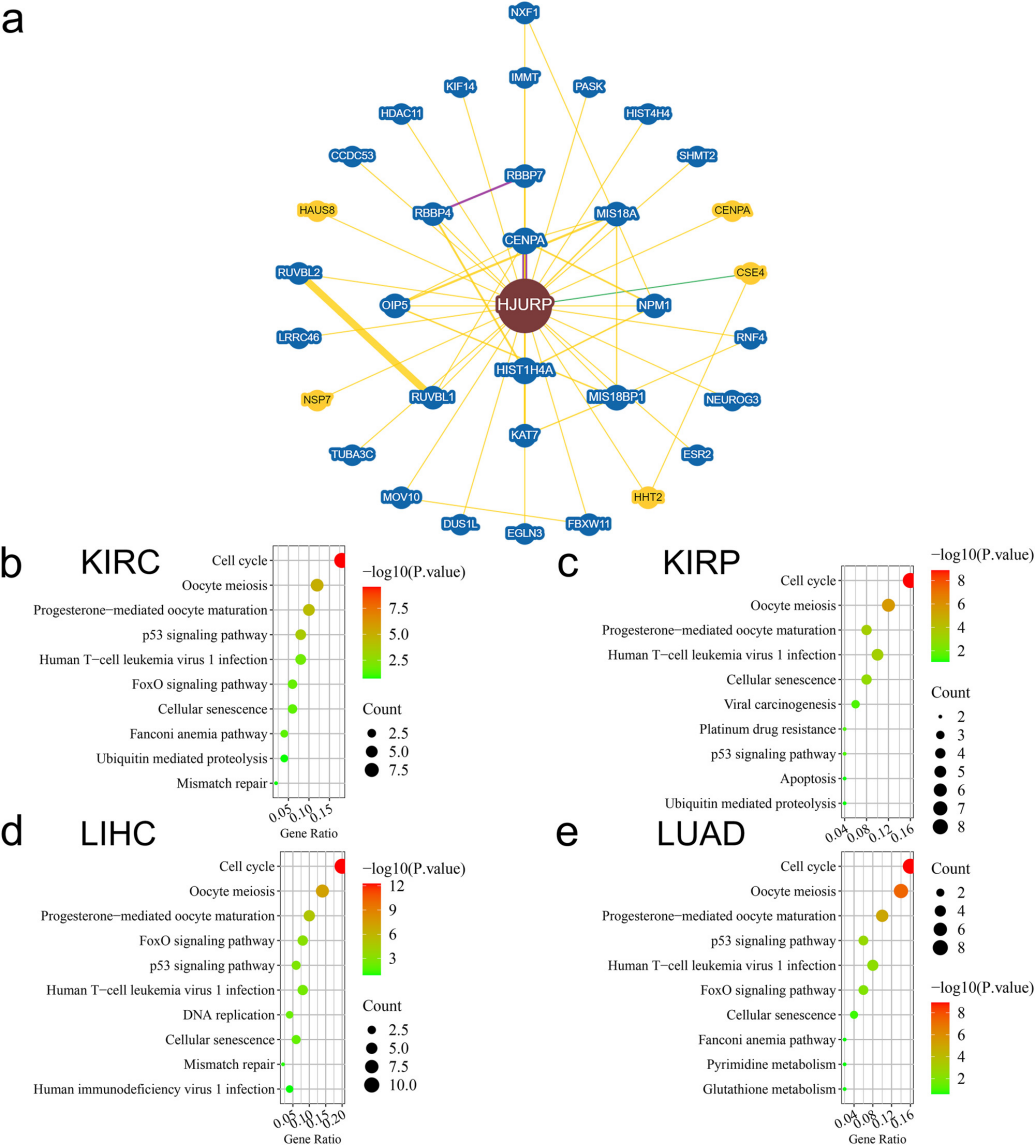
Integrated network and pathway analysis of HJURP: (a) The integrated network of HJURP. Yellow means the line is based on physical interactions. Green means the line is generated from genetic interaction. Purple means colocalization. Node size stands for its weight in the network. (b–e) Pathway enrichment of top 50 genes with the highest expression similarity to HJURP in different tumor types.

To obtain more functional insights for HJURP, an integrative network was built on coexpression, physical interaction, genetic interaction, where we found that the most related protein is CENPA, and the linkage is supported by an earlier study (Figure 4a) [16]. HJURP is the molecular chaperone of CENP-A, which is considered as an epigenetic mark of the centromere [17]. Integrated network analysis revealed that HJURP may associate with cell cycle, further affecting carcinogenesis. Based on the GEPIA database, we calculated the Pearson correlation coefficient between HJURP and all other genes among KIRC, KIRP, LIHC, and LUAD, and obtained the top 50 genes with the largest correlation coefficient. Pathway analysis was conducted based on the top 50 genes to demonstrate their molecular function and biological process by DAVID. Pathway analysis revealed that these genes converged on cell cycle and p53 signaling pathway, which are components of carcinogenesis. The top ten most related pathways are shown in Figure 4b–e.

### 3.4 Nomogram construction for HJURP based on TCGA

**Figure 5:**
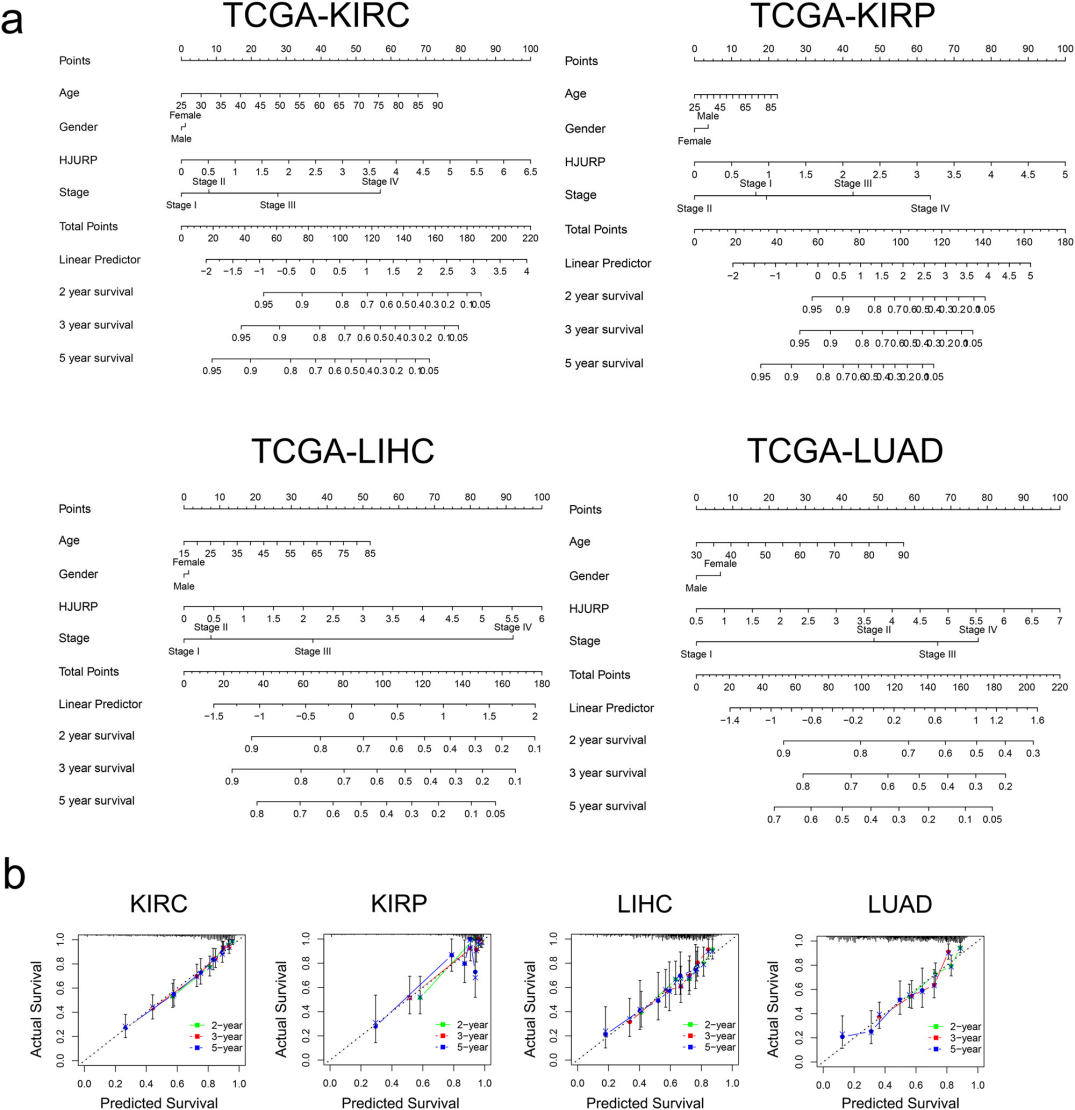
Nomogram construction and validation: (a) Nomogram for predicting 2, 3, and 5 year OS for different cancer patients based on expression of HJURP and clinicopathological parameters. (b) Calibration curves of nomograms in terms of agreement between predicted and observed 2, 3, and 5 year outcomes in TCGA cohort. The dashed line of 45° represents perfect prediction, and the actual performances of our nomogram are shown by green, red, and blue lines.

To predict the probability of cancer recurrence, we built some nomograms that combined both HJURP and clinicopathological factors (Figure 5). Researchers can use our nomograms to predict the clinical prognosis of specific individuals based on the age, gender, pathological tumor stage, and the absolute expression of HJURP (transcripts per kilobase of per million mapped reads). These nomograms of KIRC, KIRP, LIHC, and LUAD showed a good predictive power, especially for 2 year survival. The calibration plots demonstrated that the developed nomogram performed well.

## 4 Discussion

It has been reported that HJURP played an important role in human neoplasms [18]. HJURP has been demonstrated to participate in a number of biological processes including cell cycle across different cancers [19]. Moreover, HJURP exhibits some oncogenic activities in various cancer types such as breast cancer and liver cancer. Through a literature search, no reference was found with the pan-cancer assessment of HJURP of overall tumors. Thus, in this study, we examined the HJURP gene in 20 kinds of tumors based on TCGA and GEO databases and identified that the cell cycle and p53 signaling pathway triggered by HJURP are the key factors for tumor growth.

**Figure A2:**
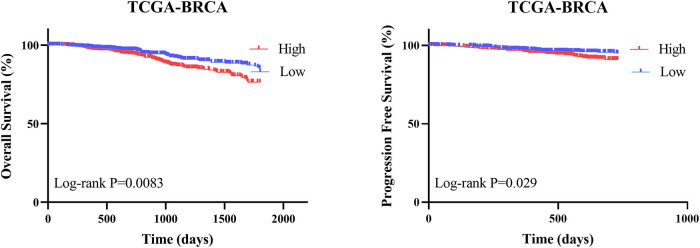
The relationship between the expression of HJURP and 5-year overall survival and 2-year progression free survival in TCGA-BRCA datasets.

Two studies have reported that HJURP mRNA contents remained an independent prognostic factor for DFS and OS [20,21]. Based on Kaplan–Meier analysis containing datasets (GSE1456, GSE2034, GSE9195, GSE11121, and GSE12276), high expression of HJURP was related to poor clinical outcomes, such as OS, distant metastasis-free survival, and relapse-free survival. Among these breast cancer cohorts, GSE1456 contained breast tumors of different molecular classifications, GSE2034 was a dataset from breast tissues from lymph node-negative patients, and the GSE12276 dataset evaluated breast cancer samples from patients who have had brain metastasis. The significant association between the expression of HJURP and the prognosis of patients revealed that HJURP was a general biomarker for breast cancer. Although we did not find a significant association between expression of HJURP and total survival in the TCGA-BRCA cohort, there was a significant association between HJURP and 5 year survival and 2 year progression-free survival (Figure A2).

For kidney-related tumors, Xu and his colleagues indicated *HJURP*, *ISG20*, and *FOXM1* as hub genes via weighted gene coexpression network analysis [22]. We here gave more evidence about the molecular mechanism of HJURP-inducing kidney-related tumors and the associations between HJURP and the prognosis of kidney-related tumors.

Even though we integrated information from different databases, there were still some limitations in this study. We did not confirm that the high level of HJURP is a byproduct of dysregulated signaling or a starting factor. The changes in DNA methylation and genetic alterations seem to suggest more hidden mechanisms of epigenetics, and biological experiments *in vitro*/*vivo* are needed to verify these findings and promote clinical utility. More research should be conducted to explore further the prospective role of HJURP in cell cycle modulation of tumorigenesis. All of the microarray and sequencing data were collected by bulk tumor tissue; thus, the nontumor cells could have introduced systematic bias. In the future, some researches with a higher resolution, such as single-cell RNA sequencing, should be performed.

Altogether, our data show a new understanding of the nature of HJURP in cancer. We demonstrate that HJURP has an oncogenic influence on pan-cancer, and high HJURP expression worsens the survival of cancer patients. Collectively, HJURP is not only a biomarker of carcinogenesis, but also a marker of poor prognosis.

## Abbreviations

ACC: adrenocortical carcinoma
BLCA: bladder urothelial carcinoma
BRCA: breast invasive carcinoma
CESC: cervical squamous cell carcinoma and endocervical adenocarcinoma
CHOL: cholangiocarcinoma
COAD: colon adenocarcinoma
DLBC: diffuse large B-cell lymphoma
ESCA: esophageal carcinoma
GBM: glioblastoma multiforme
HNSC: head and neck squamous cell carcinoma
KICH: kidney chromophobe
KIRC: kidney renal clear cell carcinoma
KIRP: kidney renal papillary cell carcinoma
LAML: acute myeloid leukemia
LGG: lower grade glioma
LIHC: liver hepatocellular carcinoma
LUAD: lung adenocarcinoma
LUSC: lung squamous cell carcinoma
MESO: mesothelioma
OV: ovarian serous cystadenocarcinoma
PADD: pancreatic adenocarcinoma
PCPG: pheochromocytoma and paraganglioma
PRAD: prostate adenocarcinoma
READ: rectum adenocarcinoma
SARC: sarcoma
SKCM: skin cutaneous melanoma
STAD: stomach adenocarcinoma
TGCT: testicular germ cell tumors
THCA: thyroid carcinoma
THYM: thymoma
UCEC: uterine corpus endometrial carcinoma
UCS: uterine carcinosarcoma
UVM: uveal melanoma
OS: overall survival
DMFS: distant metastasis-free survival
DRFS: distant recurrence free survival
DSS: disease-specific survival
RFS: relapse-free survival
